# Report of the Commissioners for Inquiring into Naval and Military Promotion and Retirement

**Published:** 1843-07-01

**Authors:** 


### Report of the Commissioners for enquiring into Naval and Military Promotion and Retirement. With Appendices. Medical Department of the Army. Presented to both Houses of Parliament by Command of Her Majesty. 1840.

In a recent number we glanced at the rise, progress, and decay of the Army Medical Board in England; and we took occasion to mention, at the same time, some of the acts of the Medical Boards in the East Indies, which, in our estimation, entitled them to a similar fate with their prototype of the mother country.

We believe it will be found with Boards, as with individuals—that they can only be seriously injured in character by their own acts of omission, or of commission; and we anticipate that we shall be admitted, by impartial persons, to have exhibited enough of the one kind, and of the other, justly to entitle both the European and the Asiatic Boards to the doom which necessarily awaited the one, and which as necessarily impends over the others.

The present admirable Director-General of the British Army Medical Department joined what was still called "the Medical Board," in 1815; and, though much the junior officer, continued the influential, and, as may well be supposed, the guiding and ruling member until 1833, when, by the natural removal of the last rags of the old system, he became the sole and responsible head of the Medical Department of the Army—a position to which his character and talents, as well as his long and tried services in the East and West Indies, in Egypt, in Walcheren, and in the Peninsula, during the campaigns of Wellington, so justly entitle him—a position in which, we will venture to say, he has not disappointed the expectations of any good man in the army, whether above or below him in station. This is great praise, we know; and we can assure the distinguished officer under consideration, as well as the public at large, that we make it as advisedly as impartially. We have no motive in stating other than what we believe, and indeed know, to be the truth.

In our former article we adduced sufficient evidence of the crying public evils attending the government of a Medical Board in London; and having done so, we shall not detain our readers with a detail of the feeble, petty bickerings—the vapid, wordy "minutes in dissent"—with which, in their last moments, and when they had lost the power of doing evil, the senior members worried and vexed the kindly-minded and excellent Chief, the Duke of York. Suffice it to say that, though they could never agree amongst themselves on any point, they nevertheless, like other old men, in other parts of the world, objected to everything, however excellent, that came from a junior, or that they could not be made to understand, or of which they could not be expected to see the end;—and thus expired, as full of age as wanting in public honour, the Army Medical Board of England.

The Report of the Commissioners* occupies but four pages of large print, and need not detain us long.

It affords, indeed, a most emphatic and melancholy instance of the cold and unpardonable indifference with which all matters connected with the medical department of the army are viewed by those even who might be expected, from previous habits of life, if not from feelings of personal gratitude, to look upon them with a friendly and fostering interest.

* Duke of Wellington,

,, Richmond,

Lord Minto,

,, Melville,

,, Howick,

,, Hill,

Sir G. Cockburn,

,, H. Hardinge,

,, James Kempt,

,, R. A. Vivian,

,, Chas. Adam,

,, A. Dickson.

"We would beg to observe to your Majesty," says the report, "that the general impression produced on our minds by the examination of the evidence, both verbal and documentary, which was placed before us, was that the condition of the army medical officers was not unfavourable; that the great advantages of increased pay and increased retired allowances which were given to this class of officers by the regulations of 1830, were well calculated to secure a succession of accomplished medical officers for the military service; and that but few changes in those regulations would be requisite to place that branch of the service in a satisfactory position in relation to other ranks in the army.

"According to the existing establishment of medical officers as laid down in the Warrant of 1830, there are six classes, viz:—

Inspector General of Hospitals,
Deputy Inspector General of Hospitals,
Assistant Inspector of Hospitals,
Staff Surgeon,
Regimental Surgeon,
Assistant Surgeon, and the course of promotion is regulated throughout upon a principle of selection.

"The Assistant Surgeon is eligible for promotion to the rank either of staff surgeon or regimental surgeon upon vacancies in the authorised establishment, while his subsequent advancement from the rank of surgeon, must, under the warrant, be made through the commission of assistant inspector.

"The Deputy Inspector Generals' appointments are filled by selection from the assistant inspectors; and the deputy inspectors furnish the candidates from whom the inspectors general are taken."

The Report then goes on to state that, while in general it approves the existing regulations, they require amendment, for the purpose of rendering more clear the demarcation of the comparative ranks and respective duties of medical officers of the different grades, and also for that of diminishing the time which elapses before they can expect to receive the first step of advancement in their profession.

"The Staff Surgeons ought, we conceive, to be considered a superior appointment to that of regimental surgeon; but this superiority can hardly be said at present to exist, since the assistant surgeon may now be appointed to be a staff surgeon, without having previously gone through the rank of regimental surgeon, while the staff surgeon, who throughout the war was accustomed equally with the physician, whose place is now filled by the assistant inspector of hospitals, to look forward to the rank of deputy inspector, as a step immediately above him, is now required to pass through the rank of assistant inspector, for which the regimental surgeon is not considered less eligible than himself."

In order that each class of officer might bear the name more correctly describing their respective situations, the Commission recommended to give to those staff assistant surgeons, whom it is proposed to place on a level with the regimental surgeon, the appellation of staff surgeon of the second class.

"The regular course of promotion in the army medical service would then be as follows:—

1. Assistant Surgeon (staff or regimental),
2. Surgeon (regimental or staff of the second class),
3. Staff Surgeon,
4. Deputy Inspector,
5. Inspector.

"The difference of pay between the regimental and the staff surgeon is also insufficient to counterbalance the advantage which the former derives from his mess and other regimental privileges, insomuch that it is practically found that regimental surgeons are frequently unwilling to accept the appointment of staff surgeon. It appears to us also that the nature of the duties which should properly belong to medical officers on the staff does not require that there should be so great a number of different ranks of these officers as at present exists."

For these reasons the Commissioners recommend the substitution of the scale of grades last given, in place of that first quoted, and the abolition consequently of the rank of assistant inspector, while the pay and comparative rank in the army now enjoyed by this class of officers should be conferred upon the staff surgeon; which appointment should be in future considered the intermediate grade between the regimental surgeon and the deputy inspector of hospitals. These suggestions of the Commissioners have, we believe, been adopted.

The conclusions submitted to Her Majesty's consideration are as follows:

"1. That it is not expedient to alter the existing rules with respect to the promotion of the medical officers of the army, for the purpose of facilitating promotion upon half-pay or by brevet.

"2. That to extend the means of promotion upon full-pay, it is expedient to make the following changes:

1st. To abolish the rank of assistant inspector.

2nd. To give to staff-surgeons the pay and half-pay, and the comparative rank with other officers of the army, now enjoyed by assistant inspectors; making also the rank of staff surgeon the regular intermediate step from that of regimental surgeon to that of deputy inspector.

3rd. To divide the assistant staff surgeons into two classes, allowing the first class the rank and pay of regimental surgeons.

"3. That it is expedient to allow medical officers retiring after twenty-five years' service, the advantage of reduced instead of retired scale of pay.

"4. That it be recommended to the military authorities of the army to consider of the means of giving to the medical staff officers attached to an army in the field, the services of a soldier servant, which shall avoid to weaken the ranks of the battalions or regiments of the army in the field.

"5. That when not employed in the field, such medical staff officers should receive, as compensation for the services of a soldier, an allowance not exceeding 1s. a day."

We now propose to analyze the evidence of Sir James M'Grigor, and of Mr. Guthrie, as given in the verbal and documentary forms to the Commissioners for inquiry into Naval and Military Promotion and Retirement.

To the question—do you experience any difficulty in finding surgeons to serve in the army either by summoning those from half-pay, or by new appointments? The Director General answered :—" I have no difficulty in finding well-qualified gentlemen, but I have prepared a statement of the difficulties which I find in carrying on the service." The difficulties set forth are:—the slowness of promotion, and 1st. That a gentleman, who, after completing an expensive medical education is about to decide whether he will enter the medical department of the army, or pursue his profession in civil life as a physician or surgeon, on looking into the regulations, will observe that after a service of five years he may obtain a step of promotion to regimental surgeon, that in two years more he may be a staff surgeon, in three more an assistant inspector of hospitals, in two more a deputy inspector, and in three more he may obtain the rank of inspector general of hospitals, so that at 39 years of age (24 being the average at which medical officers enter the service) he may attain the highest rank in the medical department of the army." How desirable soever that, for the good of the state and of the service, these rules should be acted up to, the Director General has no difficulty in shewing, statistically, how differently this inviting prospect turns out, to the disappointment and chagrin of individuals, no less than to the injury of the service, which, in consequence of the slowness of promotion amongst the assistants, " is languidly carried on."

2ndly. That, though old officers may be fit for the discharge of light duty at home, and in some of the more healthy stations, yet, in such service as that of North America at present, or tropical climates, and almost all foreign stations, they are inadequate to the duties: many of them are valuable officers with much service, discharging the duties entrusted to them to the best of their ability, but they cannot be considered as effective officers.

3rd. That this stagnation in advancement operates most injuriously in the cases of officers to be selected for the duties of superintendence on foreign stations, where active and able officers are required.

4th. That even the district surgeons at home, not to speak of the other staff officers on the full and half-pay, are old, with constitutions much impaired by previous long service in various climates, and are by no means fit again to embark for foreign service ; and this, though they are admitted to be men of intelligence, judgment, and discretion.

5th. That from a list of staff officers of the ages of 61, 64 and 65, exhibiting the actual ages of 147 staff officers on the half-pay, the Director General could not, if called on by the General Commanding in Chief, select one from the list equal to the duties of active superintendence on a foreign station. The same authority adds:—I need not to this Board dilate at any length on the important duties which devolve on superintending officers on foreign stations, and the special qualifications required of an officer for those duties ; it is sufficient to say that they ought to be active as well as able officers, with experience of service.

6th. That as to the regimental surgeons in a period of peace, particularly on foreign stations, as in India and in the West Indies, the only severe duty falls on the medical officer. In tropical and unhealthy climates, the duties of the medical officers are at all times heavy and unremitting, more particularly so from the repeated reduction in their number on every station; the consequence is, that annually, numbers of them return to this country in broken-down health, and the mortality is considerable, yet it is from these classes, as before observed, that the higher ranks of the department are to be filled up.

7th. That extreme hardship exists, in the reduction in the half-pay of each class of officers, who are so placed on half-pay on any other account than by reduction of establishment, as it bears very hard on those officers who have through great zeal and exertions, particularly in unhealthy climates, been compelled, perhaps contrary to their wishes, on the proceedings of a Board appointed to enquire into their state of health, to retire. The hardship is certainly much greater to the officer who is compelled to retire on half-pay on account of broken-down health by exertions in the service, than to the officer who is temporarily placed upon half-pay on account of reduction of establishment. In truth, the restriction of the larger half-pay to those officers only who have retired on account of reduction of establishment amounts nearly to the abolition of the larger half-pay rate, for the number of such now is almost nil.

8th. That the staff assistant surgeon is often placed in great difficulty by having no servant allowed him, and it is consequently submitted that, if he be not permitted to employ a soldier as a servant, in the same manner as the regimental assistant surgeon, an allowance be granted to him in lieu of it.

9th. That hardships attend the situation of staff surgeon, such as already remarked in the report of the Commissioners.

10th. That at the late occasion of the coronation, when the navy and army obtained promotion in all the higher ranks, the medical officers of the army were passed over. They felt this the more on seeing that the civil department of the army, the commissariat, partook of this promotion, as likewise the medical officers of the navy. Had the promotion been extended to the medical officers of the army, by providing for some old officers as in the military departments, an opportunity would have offered of bringing forward some younger and active officers more equal to the duties abroad. The promotion granted had no doubt its cheering effects on the zeal and exertions of the officers of the commissariat department, and medical officers of the navy. The exclusion of the medical officers of the army was most mortifying to them, as they felt, and I believe justly, that they had not less faithfully and zealously discharged their duties than their brother officers of the other departments of the service.

11th. That the officers of the medical department should be allowed the same prospect of promotion with the other officers of the army, military and civil, as the Director General feels confident such prospects alone can keep up the efficiency of the department, particularly in foreign and unhealthy climates, where so much is expected from them, and where the efficiency of any force so much depends upon their zeal and exertions; and the Director General feels warranted in offering his humble but decided testimony to the advantage that would accrue to the service of the public, by showing to the whole department, by some general and liberal act of favour, that in a period of peace their services were estimated by the Government of not less value than in the period of war, when he, the Director General, was proud to say their services received repeatedly the most gratifying public approbation, followed by promotion.

Such are some of the difficulties experienced by Sir James M'Grigor "in carrying on the service." It will be perceived that they consist principally in acts of neglect and injustice, inflicted on those under his orders, by the government of the country. We have no right to expect that men will go eagerly to work in carrying on any service while smarting under disabilities, slights, and injuries; and, having the good of the state, of the soldier, and of the department warmly at heart, the Director General anxiously desired to secure the good will, and, by consequence, the best energies of those under his orders, by procuring for them something like an equality of honour and reward with their military brethren, to whom he felt they stood in no respect inferior, either in zeal, usefulness, or talent.

But we must now turn to Mr. Guthrie, who likewise urged the same subjects on the Commissioners, with great energy and truth. Mr. Guthrie considers:—

1st. That the refusal of promotion by brevet, on the two last occasions, is considered by the medical department an unkindness which they consider to be undeserved, and which it is believed cannot be maintained on fair investigation, with justice either to the individuals or to the public.

2nd. That, though of late the promotion and allowances of the officers of the department have been settled by Sir Henry Hardinge in a fair and reasonable manner, still the questions of widows' pension and prize money are in the same unequal state as before.

3rd. That, in order to understand the subject of promotion as bearing on the utility of the department, it is proper to revert to the objects for which it was formed, and to compare its present with its former state.

The objects of a medical department ought to be attained in the great advantages the sick and wounded may derive from the services of its officers, and the public from the improvements they may make in their profession; but, if we look back to former periods, it will be found that these objects were in a great measure overlooked, or made subservient to the wishes and interests of individuals.

Staff physicians and surgeons were appointed without having served a single day. They were taken from the universities or hospitals to learn their profession at the expense and great inconvenience of the unfortunate soldiers committed to their care. Placed at once, from their appointments, in situations of trust, they soon, if they remained in the service, claimed and obtained others: and thus, gentlemen who had little knowledge of the habits, of the duties of a soldier, of the composition of an army, or of anything else belonging to it, were to be the advisers of a general commanding in chief, who, in the field, had rather to teach them their duty than to receive their assistance. Sickness and loss of life, from their inexperience as well as from disease itself, soon rendered an army inefficient, and diminished its numbers more effectually than the enemy. It would be too personal to allude to gentlemen who, after a lapse of nearly half a century, receive large half-pay for little duty or service.

4th. That, whilst the army received little advantage from the services of those officers, the profession at large and the country benefited nothing. They did little or no work, made no improvement, and slighted their hard-working brethren, who had no interest to aid in their advancement. If the annals of surgery be consulted, no records will, I believe, be found of improvements made during the campaigns in the American war, nor in those of Holland, nor in Flanders, nor even at the Helder; and the contempt of the surgeons in civil life was not unfairly bestowed on their negligent military brethren. The fault was not alone theirs, but of the higher authorities, who either denied them the encouragement, or bestowed it wantonly or carelessly on individuals who had more or less private influence.

5th. That, during the Peninsular war, the hardships and sufferings of the medical officers became so great, that the government could not find men qualified to serve in the junior ranks who would go to that army. It would naturally be supposed that the course to be pursued in such emergency would have been to remove the grievances and defects complained of, or to have increased the rewards. A different plan was, however, adopted; and, as competent or commissioned officers could not be found willing to serve, it was thought right to send incompetent men as warrant officers, hired for the occasion, in order to make up in quantity for deficiency in quality. I have elsewhere pointed out the destruction of human life committed by one of these persons, under this authority; and many others did the same, without, however, any real blame attaching to them, for it was known to those who sent them that they were for the most part ignorant of the profession of physic or surgery.

6th. That there were, however, in that army, many officers who did their duty well, and who, whilst their military brethren raised its renown to the highest pitch of reputation and character army ever attained, formed for it also a surgical reputation which has not been excelled by that of any nation in Europe. They have left precepts and records which have been since followed and tried on several of the same fields in Spain, with the greatest advantage. They have the happiness of knowing that they have been the means of preventing and of alleviating much human suffering and misery, of saving great numbers of lives, and of originating one half of the improvements which took place in surgery during that eventful period.

7th. That many of these officers, who have served on full-pay nearly ever since that time, have nevertheless the misfortune to find that they alone of the whole army have latterly been overlooked and considered unworthy of any marks of approbation, and the additional mortification of seeing that this neglect has not been applied to their contemporaries in foreign armies, nor to themselves when employed in civil service, nor to their brethren in civil life.

8th. That it is considered right and proper, and worthy of a great country, as it assuredly is, to reward the exertions and energies of sailors and soldiers by promotion, by honors, and by pensions, long after the time when these exertions and these energies have been called forth; and it is honourably due in grateful and just remembrance of the services. It is only to the surgeons of the army that promotion or honors, or pensions granted in a similar manner and for similar services, are considered unnecessary, and are absolutely refused.

9th. That, when worn out by long service, they are allowed to retire on a reasonable allowance, like a private soldier, but without anything, even as in his case, which can distinguish the best and the most scientific from the worst and most negligent officers in the service. The medical officers in the army may then be said to be governed at present by the system of punishments rather than of rewards—a mode of proceeding that has always been unsatisfactory to all parties, has usually failed, and in the present day has been frequently reprobated by many enlightened men.

10th. That it is very humbly submitted that, to preserve to the service a continuation of that scientific and practical improvement which has been so remarkable since the commencement of the last war, when compared with that which preceded it, promotion in the medical department should take place in a manner similar, or nearly similar, to that in which it is granted to the army at large, by fixed and not by arbitrary rules; and that, as medical men are human beings, subjected to the same influences, feelings, and passions as other men, they should have held out to them, at all times, the same or similar inducements to good conduct, to exertion, and to emulation in their profession, as are found, or are supposed to be necessary or proper for the officers of the army at large.

Such are, almost ad verbum, the sentiments placed on record by Sir James M'Grigor and Mr. Guthrie.

When required to state any plan which may have occurred to him with a view to relieve the grievances complained of, Mr. Guthrie states that the first grievance demanding serious consideration is, the slowness of promotion. He would recommend that no assistant surgeon be allowed half-pay till he had been seven years in the service;—that after five years' service he should be allowed a gratuity of one year's pay, and of two years' pay in event of his remaining near seven years in the service: that, in event of his retiring from ill health, he should have a half-pay of four shillings; but, if retiring on reduction, or from other authorized cause, a half-pay of three shillings should be allowed after seven years' service:—that an assistant surgeon should not be allowed to serve as such beyond twelve years, or fourteen years, under any circumstances:—that, after fourteen years' service he should have the unqualified right to retire permanently with the rank of unattached regimental surgeon, and the half-pay of six shillings, or seven shillings a day; because, if, between 14 and 20 years, he has not been promoted to the rank of surgeon, it must be from some defect of character, and therefore it would be advisable to part with him kindly and considerately, so as to prevent his being a clog on promotion:—that, if such an officer were allowed to remain in the service up to 30 years, his half-pay would amount to 15s. a sum much beyond what he is worth:* that, in regard to regimental surgeons, who are now permitted to retire after 25 years on 11s. 6d. and after 30 years on 15s. per diem, the regulation be altered, so as to allow the latter half-pay, to 25 years' service, seeing that an officer of 30 years standing must, comparatively speaking, be an inefficient officer, averaging 54 years of age:—that by such an alteration in the rules the public would neither gain nor lose much,

* "Now the difference between 7s. and 15s. is a great deal more than he is worth, if he is not equal to all kinds of duty in every climate, and consequently the public will do well to part with him after twenty years of service at 7s. a day."

whereas the sick and wounded soldier would be much benefited :—that, in short, every practicable inducement be held forth to old officers to retire, so as to bring forward their younger and more effective brethren ;—that after 25 years he would never allow a regimental surgeon to be promoted to a staff surgeon, because whenever an officer is promoted after that time, it is generally for his own convenience, and not for the advantage of the service :—that when gentlemen have served 30 years, a great change has taken place in their professional prospects ; and being too old for exertion in civil life they are reduced, when on half pay, to live like other officers, the best way they can, their profession being of little use to them :—that such officers, when of good character, should be Gazetted out with the rank of unattached staff surgeon :—that as to the staff surgeon, he is the most important officer in the service, and the one in whose situation the greatest change ought to be made, because he is the officer on whom everything depends, either at a station or with a division in the field :—he should superintend and govern the regimental surgeons, look to such irregularities in the internal management of corps as might lead to disease, attend to all points as regards clothing, quarters, diet, and the general position with respect to salubrity; and no sick man should leave the division without his permission :—that to do all this, his rank should be above that of regimental surgeon :—that commanding officers should be in the habit of looking on the staff surgeon as a superior officer of character and reputation in his profession, the arbiter of their lives and limbs, for it is on his knowledge and decision that their lives and limbs will, in the hour of need, depend :—that he should be promoted by selection from among the regimental surgeons, that he should not be placed on the staff till he had been eight or nine years in the service, and have gone through the respective grades of assistant surgeon, and surgeon, so as to be thoroughly acquainted with the management of a regiment :—that the office of assistant inspector be abolished, giving his rank to the staff surgeon, with enhanced pay :—that formerly, when an army took the field, it was supposed it ought to have a surgeon who knew little or nothing of physic, and a physician who knew nothing of surgery ;. but of late years the education of all medical officers has been made the same, and therefore, when the chief of a staff goes on service, he may say, I have three staff surgeons who are addicted to physic and three who have an inclination for surgery, therefore to the hospital that contains only sick I shall send the staff surgeon who is addicted to physic, and to the other hospital which contains wounded, the staff surgeon who likes the practice of surgery ; but as a battle is an occasional thing and sickness is always present, they should be equal on every point :—that the staff surgeon is both physician and surgeon, and should be selected from the regimental surgeons for his ability and for nothing else, which must be done by the head of the department : —that in respect to the deputy inspector of hospitals, he should be selected precisely in the same way, from the staff surgeons of about twelve years standing in the service, and for his ability and talent :—that the staff surgeon should retire after thirty years' service, in the same manner as the regimental surgeon ; but he should be Gazetted with the rank of deputy inspector general of hospitals :—that he should not again be employed as a staff surgeon, although, if not too old, he may be employed as a deputy inspector, that office being one principally administrative and not practical: —that an officer should never be promoted by brevet and remain in the same situation he was in before:—that the pay and all other allowances of deputy inspector should be settled as equal to those of the corresponding rank of lieutenant-colonel on the permanent staff of the quarter-master general, and that their widows should also be put on a perfect equality:— that, in respect to the rank of inspector general, there is an essential grievance:—that he ought to be promoted in two ways, by merit, when conspicuous or pre-eminent, and also by brevet, according to his standing with lieutenant-colonels of the army, that is, he should have the rank of inspector:—that to encourage promotion the inspector be required to retire after four or six years—in short, that the inspectorial offices should follow the rule which govern generals, admirals, and governors in like cases:—that deputy inspectors should be promoted by brevet, without pay, according to their standing with lieutenant-colonels, so as to confer on them the much desired rank of inspector, and to which they are fairly entitled:—that on the promotion of the deputy inspectors to the rank of inspector, the vacancies made by such promotion to be filled up by selection from the staff surgeons:—that the brevet promotion of deputy inspector should be by seniority, as in the army, but promotion to those below, when they make vacancies on service, to be done by selection:—that, when made an inspector, it is not necessary to employ him as such unless well fitted for the duty, the rank being conferred in reasonable compensation for long service: —that as such officers are not likely to get any private practice, it is well to let them go into society at large with a proper addition to their rank, which costs nothing; and therefore it is that I would Gazette the staff surgeon out as deputy inspector, and the deputy as an inspector general: that the rules relating to the distribution of prize-money should be made of equal operation.

Mr. Guthrie concludes his testimony to the importance of his department and to the claims and merits of his brother officers, by an appeal to the Commission on the subject of "rewards and honours"—a subject which has not met with the attention it deserves, from its not being the particular duty of any one person to bring the services of medical officers under consideration in the proper quarter, and not, Mr. Guthrie believes, from their labours being unappreciated, or from deficiency of good feeling towards them on the part of the higher authorities, or from disinclination on the part of the Sovereign, as the fountain of all honour, to bestow such acts of grace and favour upon them.

"It is probable that the peculiarity of not bestowing rewards on military medical men, for services in the field, has arisen from their being considered and called non-combattants; but nothing can be more unreasonable than the classification of medical officers with clergymen and other civilians attached to the army, with reference to their being exempt from danger; for it is well known they are always exposed to it in the field, and many have been killed and wounded. When an army is engaged in position, surgeons may generally get under cover, but when an army advances to attack, or, when attacked, is obliged to change its position, every medical officer must move with his regiment, until he is arrested by his wounded. I am aware it may be said, that surgeons should not be so exposed, but every one who has been a regimental officer knows well, how often the surgeon is called to the front, even when a single man is wounded.

The soldier expects this assistance, and medical men have never disappointed him, neither in the French nor the British armies. In a professional point of view, this close attendance has often been the means of saving life, and an examination at this, the earliest possible period, has often saved a limb. In every siege a medical officer is always sent into the gorge of the trenches, and they have at all assaults, marched with the troops. They are thus, in fact, exposed to a great part of the dangers of the field, and afterwards to those of their own profession, which crowded hospitals engender, and which have often been most fatal. In both instances they are obliged to shew a degree of moral courage and devotion which, I humbly submit, will be greatly encouraged by a little favourable consideration. In all foreign armies this consideration is given by a liberal distribution of rewards and honours which are of great importance to the individuals. In London it matters little whether a man is an esquire, a cross, or a baronet, the public take him as they want him, and for what he is worth; but in the country and abroad, where persons are more in contact, it is very different; and a great deal of attention is paid to a man who can shew, by his rewards and honors, that he has a fair pretension to merit gained on service. In the French army, I am expressly informed by the Baron Larrey, that Napoleon conferred on Desgenettes, Percy, and himself, in 1804, the gold cross, or that of an officer of the Legion of Honour; in 1807, that of commander; and, after the battle of Wagram, he bestowed upon them and several others the commandery of the Iron Crown and the rank of Baron, promoting Larrey from the surgeoncy of the Imperial Guard to the rank of Inspector General, although he was never the administrative head of the medical department of the French armies. In this way encouraging and rewarding those improvements in science, and those efforts made in the cause of humanity, which have rendered those gentlemen conspicuous throughout Europe. I am not aware that the surgeons of the French army have had a greater claim on account of their services, on their sovereign or their country, than those of the British army. We consider ourselves to have been equal to them in all respects; and, if it were necessary that either class of men should shew one small step in advance of the other, I am prepared to prove that that small step—(tant soit peu, as it may be) is not against us. In order to remedy the grievance complained of, I would submit the two following modes of proceeding for consideration.

"1st. That officers who have served on full pay for 25 or 30 years, and have attained the rank of inspector general, should be permitted to submit their claims to the Commander in Chief, for the honour of Knighthood, and, if he should approve the appointment, to be Gazetted as at his recommendation.

"2nd. That all medical officers serving in future in the field, and actually before the enemy, should share in the rewards and honours granted to the army at large, according to their respective and relative ranks, and under the same regulations.

"I would very submissively beg leave to remark, in conclusion, that in advocating the case of the older medical officers of the army, I have had also a much higher object in view, viz. the relief of the sick and wounded soldier, in future wars, from those distresses, miseries, and horrors, which, during the last war, the unbounded care, the attention, and the paternal kindness of the Duke of Wellington could not entirely prevent or remove; and which can only be alleviated or removed through the medium of medical officers of good administrative knowledge, and the best professional ability.

"Such men can only be obtained by adequate remuneration, rewards, and honours."

We come now to the Rules and Regulations under which the medical officers of the army are appointed, promoted, and permitted to retire from the service; and we find that, on the appointment to office of the present

Director General, he received from the Commander-in-Chief the following instructions:—

"All promotions and appointments in the medical department of the army, to be recommended by you to the Commander-in-Chief.

"You will in your recommendations for promotion be governed by the usual rule of seniority, as far as in your judgment circumstances will permit.

"You will select and submit for the Commander-in-Chief's approbation all officers and persons to be employed in the medical department abroad, and at home."

On these instructions I have acted since I had the honor to be appointed to the office I now hold.

A statement is inclosed of the qualifications required of every candidate for appointment to the medical department of the army, which further gives the age at which they are admissible to the service. I find, however, the actual age of those admitted to be between 23 and 24 years.

When a candidate is found to possess the required qualifications, his name with a detail of his education and qualifications are entered at length in the book of candidates, and in submitting names to the Commander-in-Chief, the only preference given beyond the standing in the book, is to those of very high qualifications, and to the sons or orphans of military or medical officers, who have suffered grievously in the service, but the name of no candidate is submitted who does not possess the required qualifications.

Every candidate is examined by a board of medical officers at which I preside, and it is required that his diplomas, all vouchers of qualifications, certificates of the different professors and lecturers, of regular attendance, and likewise certificates of moral character and conduct, be deposited in my office ten days before the day of examination for inspection and examination. Unless by the proceedings of a Court of Inquiry, or of a Court Martial, there appears anything to warrant an officer being passed over, I invariably adopt the rule of seniority in the service in recommending the promotion of assistant surgeons to regimental surgeoncies, of regimental surgeons to staff surgeons, and of staff surgeons to be assistant inspectors of hospitals; but in recommending to the two higher ranks, viz. those of deputy inspector general and inspector general of hospitals, I find it expedient to adopt a different line. As the officers of these two classes are for general and extensive superintendence on foreign stations, I think it right to make a selection, and to recommend (from the knowledge I possess of the merits and talents of all), an officer who unites with a thorough knowledge of the service, and of the professional duties, talent for arrangement and habits of business, together with discretion, discernment, and conciliatory manners, and who can, from his character in the service, command the respect of those acting under him.

A copy of the warrant of 29th July, 1830 (Paper B), which is annexed, will explain the course of promotion in the medical department of the army, and show the rates of full pay; and the extracts from the Warrant of 22nd July, 1830, (Paper C), will show the rates of half-pay.

(Signed) J. Mc Grigor, Director General.

I ————————*, ——— years of age, a candidate for employment in the medical department of the army, do hereby attest my readiness to engage for general service, whether at home or abroad, and to proceed on duty immediately on being Gazetted.

I declare my age not to exceed twenty-six years, that I am unmarried, and that I labour under no mental nor constitutional disease, nor physical debility, that can interfere with the most efficient discharge of the duties of a medical officer.

(Signature.)

* Christian and surname at full length.

I have pursued the undermentioned course of study, of which I am ready to produce the vouchers for registry, and also a certificate of my age; namely :—

I possess certificates of regular attendance of the undermentioned hospitals and courses of lectures for the number of months stated :—

| | | |
|---|---|---|
| The ——— | Hospital or Infirmary for | Months. |
| The ——— | Hospital for Mental Derangement for | ,, |
| The ——— | Hospital for Diseases of the Eye for | ,, |
| The ——— | Lying-in Hospital for | ,, |

| Lectures. | Professor's Names. | Place. | Period in months. |
|---|---|---|---|
| Anatomy and Physiology, by .. .. (Stating the number of Lectures of each course on separate days.) | | | |
| Practical Anatomy, by . .. .. .. (Stating number of subjects dissected.) | | | |
| Stating the number of Lectures in each course. | | | |
| Surgery, by.. .. .. .. .. .. | | | |
| Clinical Surgery, by .. .. .. | | | |
| Military Surgery, by .. .. .. | | | |
| Institutes of Medicine, by.. .. | | | |
| Practice of Medicine or General Pathology, by.. .. .. .. | | | |
| Clinical Lectures on the Practice of Physic, by .. .. .. .. | | | |
| Midwifery, by .. .. .. .. | | | |
| Chemistry, by .. .. .. .. | | | |
| Practical Chemistry, by . .. | | | |
| Botany, by .. .. .. .. .. | | | |
| Materia Medica, by .. .. .. | | | |
| Practical Pharmacy or an Apprenticeship .. .. .. .. | | | |
| Forensic Medicine, by .. .. | | | |
| Morbid Anatomy and Pathology, by .. .. .. .. .. .. | | | |
| Natural History, by .. .. .. | | | |
| Natural Philosophy, by .. .. | | | |
| Moral Philosophy, by.. .. . | | | |
| Mathematics, by.. .. .. .. | | | |

I have the Degree of A. M. or A.B. from the University of
I have the Degree of M.D. from the University of
I have a Diploma for Surgery from the College of Surgeons of
(Signature.)
(Date.)
(Place of residence.)

* The date of Graduation to be stated.

1st. In selecting from among the candidates for the medical department of the army, a preference is given to those who can fill up all the blanks in the preceding pages, by having the acquirements there stated, but the name of no gentleman can be placed on the list who does not possess the Diploma of either of the Colleges of Surgeons of London, Edinburgh, or Dublin, and who cannot produce the following testimonials :—

| | | |
|---|---|---|
| 18 | Months | attendance on an hospital of celebrity, where the average number of in-patients is not less than 80. |
| 24 | ,, | Anatomy. |
| 12 | ,, | Practical Anatomy. |
| 12 | ,, | Surgery, or (what is preferred) six month's Surgery and six month's Military Surgery. |
| 6 | ,, | Clinical Surgery, a complete course of two or three lectures during the week. |
| 12 | ,, | Practice of Physic, or six months' Practice of Physic and six months' General Pathology. |
| 6 | ,, | Clinical Lectures on ditto, the same as required in surgery, |
| 12 | ,, | Chemistry. |
| 6 | ,, | Practical Chemistry. |
| 3 | ,, | Botany. |
| 4 | ,, | Materia Medica. |
| 3 | ,, | Practical Pharmacy or Apprenticeship. |
| 5 | ,, | Natural History. |
| 4 | ,, | Midwifery. |

2nd. The candidates must be unmarried, not beyond 26 years of age, nor under 21 years.

3rd. Candidates who have had an University education, and have the degree of A.M. as well as that of M.D. will be preferred, but a liberal education, and a competent knowledge of the Greek and Latin languages are indispensably requisite in every candidate.

4th. The greater the attainments of the candidates in various branches of science, in addition to competent professional knowledge, the more eligible will they subsequently be deemed for promotion in the service; for selections to fill vacancies will be guided more by reference to such acquirements than to mere seniority.

5th. The rank of physician to the forces, or assistant inspector of hospitals, requires, in addition to the knowledge and experience to be gained in the regular progress of study and experience in the service, that the individual should be a fellow or licentiate of the Royal College of Physicians, of London, or a graduate of the University of Oxford, Cambridge, Edinburgh, Dublin, Glasgow, Aberdeen, or of the Faculty of Medicine of Glasgow.

6th. Although the British schools are specified, it is to be understood that candidates who have received regular education in approved foreign universities or schools, will be admitted to examination.

7th. With the exception of Practice of Physic and Clinical Medicine by one teacher, candidates must have attended separate lectures for each branch of science.

8th. Before promotion from the rank of assistant surgeon to any higher rank, every gentleman must be prepared for such other examination as may be ordered before a Board of Medical Officers.

9th. Diplomas, tickets of attendance on lectures, and certificates of regular attendance by each professor or lecturer, must be lodged at this office for examination and registry at least one week before the candidate appears for examination, and likewise certificates of moral conduct and character, one of them by a clergyman, and that of the parochial minister is desirable. Baptismal certificates are required at the same time; if the parish register cannot be resorted to, an affidavit from one of the parents, or some person who can attest the fact, will be accepted.

10th. The certificate of the teacher of Practical Anatomy must state the number of subjects or parts dissected by the pupil.

11th. Certificates of lectures and attendance must be from physicians or surgeons of the recognised Colleges of Physicians and Surgeons in the United Kingdom, or of foreign Universities.

According to the Royal Warrant 29th July, 1830, the following regulations were put in force: * * * *

*Assistant Surgeon.*—No medical candidate who has not passed his examination at the Royal College of Surgeons in London, Edinburgh, or Dublin, shall be eligible for this commission; and the assistant surgeon must have served on full-pay five years before he shall be eligible for promotion to the rank of regimental surgeon; and seven years before he shall be eligible for the rank of staff surgeon.

5. Regimental surgeons and staff surgeons must have served ten years in the army upon full-pay, before they shall be eligible for the next step of rank.

6. An assistant inspector of hospitals must have served three years at home, and two years abroad, in this rank before he shall be eligible for promotion.

7. A deputy inspector general of hospitals must have served five years at home, and three years abroad in this rank, before he shall be eligible for promotion to the highest rank of inspector general.

8. The rates of daily pay for the before-mentioned ranks are in future to be regulated by the length of time which the officers of each class shall have served upon full-pay according to the annexed scale; provided always, that when an officer is hereafter promoted, he shall commence upon the minimum pay of his new rank, notwithstanding his length of service, agreeably to the said scale, may give him a claim to a higher rate of pay, as before he shall be allowed such higher rate of pay, he will be required to serve on each inferior rate of pay attached to his rank the following period, viz. one year, if he had been in the medical department antecedently to the date of this warrant, and two years if he received his first medical commission subsequently to that date. But if the officer thus promoted had higher pay in his old rank than the minimum of his new rank, he shall commence upon that rate of pay, which may be next above his former pay, and before he obtains any further increase, shall serve the period above prescribed, viz :—

| RANKS. | Rates of daily pay, subject to the above provisions. | | | | | | | | | | | |
|---|---|---|---|---|---|---|---|---|---|---|---|---|
| | After 25 years' Actual Service | | | After 20, but under 25 years Actual Service | | | After 10, but under 20 years' Actual Service | | | Under 10 years Actual Service | | |
| | £. | s. | d. | £. | s. | d. | £. | s. | d. | £. | s. | d. |
| Assistant Surgeon .. .. | 0 | 10 | 0 | 0 | 10 | 0 | 0 | 10 | 0 | 0 | 7 | 6 |
| Regimental Surgeon.. .. | 1 | 2 | 0 | 0 | 19 | 0 | 0 | 15 | 0 | 0 | 13 | 0 |
| Staff Surgeon .. .. .. | 1 | 3 | 0 | 1 | 0 | 0 | 0 | 16 | 0 | 0 | 14 | 0 |
| Assistant Inspector .. .. | 1 | 4 | 0 | 1 | 2 | 0 | 0 | 19 | 0 | | | |
| Deputy Inspector General | 1 | 10 | 0 | 1 | 8 | 0 | 1 | 4 | 0 | | | |
| Inspector General .. .. | 2 | 0 | 0 | 1 | 18 | 0 | 1 | 16 | 0 | | | |

In addition to the pay of their ranks, the officers at the head of the medical department on foreign stations, shall receive allowances at the undermentioned rates, when serving under the following circumstances, viz. :—

If with an army in the field of 10,000 men or upwards 20s. a day.
Ditto Ditto 5,000 men ,, 15s. ,,
Ditto Ditto any less number 10s. ,,

If serving in a colony where the forces consist of 1,500 men or upwards, 5s. a day.

By His Majesty's command,
(Signed) H. HARDINGE.

Having finished our analysis of the Report of the Commissioners, and exhibited to our readers the noble exertions made in behalf of their brethren by the Director General and by Mr. Guthrie, we would offer a few concluding remarks of our own—adding the testimony of Mr. Annesley as to the state of the Medical Establishments of India.

1. Parliamentary Reports may be referred to and consulted by persons residing in England, or by officers returning from abroad; but to the greater proportion of naval and military surgeons—to the entire body of the medical service of India—these ponderous volumes with their valuable contents, are a sealed book. We feel therefore that, in presenting an analytic view of the Report of 1808, in a recent number, as well as of that of 1840, in our present issue, we do a welcome service to hundreds of our brethren abroad.

2. On the vital question of promotion, we would call especial attention to the fact that, in the scheme of promotion for the medical department of the British army, the officers of which are presumed on an average to pass more that half their period of service in temperate climates, it is intended that a man shall attain to the highest rank in fifteen years, or at the age of thirty-nine. It is now hardly necessary to contrast such a plan of promotion—a plan urged by practical and experienced men, who have passed their lives with the soldier, in every climate, and in every situation, whether of peace or of war—with one of seniority—a plan which excludes the ablest officers from rising to the same grade, till, by a service of forty years on an average, they shall have been worn out, and attained to about the sixty-fourth year of age.

3. If it be complained of in England as a most serious evil that, owing to the half-pay list, and other causes, which we hope are but temporary, promotion to the higher staff offices has been hitherto deferred to middle life, what are we to think of a system which, in India, prescribes a still more advanced age to the same responsible stations?

4. We would call the utmost attention to the evidences regarding the most important grade of staff surgeon; and we would ask, how in any country or in any service—how, least of all, in a seniority service, and in the climate of the East Indies—how such an officer as Mr. Guthrie describes the staff surgeon, is to be taken on the seniority principle, just because he stands at the head of the muster-roll?

5. What are too commonly and very carelessly called "experience," and "service," must not be confounded with "merit."

A man may serve for half a century, and yet neglect the opportunity of observing, or he may be incapable of observing. The mere "experience" and "service" of being borne so many years on the muster-roll is therefore emphatically and justly disallowed by both Sir James M'Grigor, and by Mr. Guthrie. Yet, for the respectable officer of long standing, the brevet by seniority, and the pension for length of service, must be allowed to be both just and proper; while, at the same time, promotion by selection, in the instance of "merit," is equally necessary to the good of the service, as well as to the encouragement of men of talent.

6. The permanent establishment, however, of the two modes of promotion, just described, will, in the estimation of Mr. Guthrie, be of little avail, unless the regulated and limited tour of service in the several grades be rigorously enforced without exception, on ordinary service—at home as well as in the distant possessions of the empire.

7. On the subject of brevet, the conduct of the Commission is strange and contradictory in itself, and at variance with the most complete detail in proof, as given in evidence before the Commissioners. They declare it "not expedient to alter the existing rules with respect to the promotion of medical officers of the army, for the purpose of facilitating promotion upon half-pay or by brevet;" and they conclude by conferring brevet on the navy, which never had it, and by taking it away from the army, which partially enjoyed it since 1817.

We regret to say that this is not the only instance in which the "Report" is at direct variance with the best proof in evidence. The Commission* of 1808 performed its duty in an upright and honourable manner, while that of 1840 took but a contracted and an unkind view of the important matters under investigation. It is curious to observe that a Commission composed of gentlemen in civil life, Members of Parliament, to every one

* J. Drinkwater,

Sam. C. Cox,

Giles Templeman,

Henry Peters,

Chas. Bosanquet,

B. C. Stephenson,

L. Bradshaw.

of whom "the subject of inquiry was new," should nevertheless have investigated and opened out the entire matter so fully, as to leave it impossible for the Government any longer to continue the jobs and abuses of the Medical Board, whilst another Commission, comprising the greatest of our surviving naval and military commanders, should have done worse than nothing. But such we shall ever find it.

It was said long ago by Robert Jackson that, we had no hope but from the legislative authority, and we would do well to remember a fact often proved in the honourable endeavours of one of the most able and honest of our body. Pitt knew of the existence of enormous abuses, and remained silent, as did the military authorities; but the Commission of 1808, be it remembered again, declared and proved the "most essential measure" to be the "discontinuance of the present Army Medical Board"—a measure, we venture to say, we have proved more than once to be quite as "essential" in the East Indies in the present day.

8. Strange as it may appear, we have not a warm friend in the highest military place. It would seem that, to make sure friends for us, our commanders must be shot, and that frequently.

Out of what, in the British Army, is called the heroic school of Abercrombie, we have at all times numbered the ablest and the most powerful friends, such as Moore, Hope, Graham, and others. But it is not in the nature of things that men of such a school should be long-lived; and in Moore especially we lost our ablest and best friend. The renowned Chief who at present rules the military affairs of England—the Chief whom no one, now-a-days, ventures to gainsay, whether he be in the right or in the wrong—never chanced to have had a shot to pass either through his body, or through the long bones; and, as he is not likely to adopt, either the profound views of Napoleon, or the friendly generous sentiments of Abercrombie and Moore, and cannot see that in treating us as a caste, separate from the army, he is doing the public service an injury, and us an injustice; matters, we fear, will remain as they are for the present. But, joking apart, it is really thus with us; and nothing else stands in our way, or has deprived us of the brevet, to which we are so clearly entitled, or of the military honours, which are as clearly our due.

9. We would once more call the attention of our military brethren to paragraphs 2, 4, 5 and 11 of Sir James M'Grigor's evidence; and to the declarations of Mr. Guthrie, that, under the old administration of the "Board," the very profession of military surgeon fell into contempt, while the army, the country, and the profession at large "benefited nothing;"—indeed, the loss of life from the inefficiency and inexperience of all classes,—the choosers and the chosen—diminished the numbers of the army "more effectually than the enemy." On the other hand, under a system of judicious selection of military surgeons, Mr. Guthrie had "the happiness of knowing that they," his brethren, "have been the means of preventing and of alleviating much human suffering and misery, of saving great numbers of lives, and of originating one half of the improvements which took place in surgery during that eventful period,"—that of the Peninsular war, when men like Mr. Guthrie forced their way to eminence through the dregs of the old system, and raised "a surgical reputation which has not been excelled by that of any nation in Europe."

We would in like manner again call attention to paragraphs 9 and 10 as well as to the whole analysis of Mr. Guthrie's evidence.

10. It is no less curious than interesting and important to find that, while in Europe the noble profession of military surgeon was thus brought into general contempt, owing solely to mal-administration, the same result was brought about by exactly similar means in the East Indies. Mr. Annesley states :—that it is quite impossible the neglected and degraded state of the medical profession in India can have escaped the notice of the most common observer :—that he has long been of opinion that so lamentable a state of things can only have arisen from inefficient administration, caused by a seniority system of promotion—a system, he regrets to say, which has infested the service from the earliest times—even before the institution of Boards in 1786 :—that in these times too, a very inferior class of officers were admitted into the service, and gave to it a low tone :—that though there were, at the same time, some individuals of high character and repute, the bad and inferior vastly prevailed :—that therefore, it is no matter of surprise to find a department that ought, from its paramount importance to the welfare of armies, to stand high in order and estimation, suffered the most serious deterioration :—that, on the other hand, it is matter of wonder how a Government, which could not be ignorant of the defects of the service, should nevertheless, and with such materials, have adopted a system of promotion and administration, for the medical department, at variance with every other department of the service ; for instance, the members of council, of military boards, of boards of revenue, and of trade, have ever been selected officers :—that seniority was never thought of but in that department alone wherein it was calculated to do most injury to the public service :—that a system which takes for the control and management of a great department, three men, just as they stand in seniority order—no matter what their character or qualification—cannot be otherwise than bad, as it is quite impossible that any number of men taken consecutively, shall possess the professional character, energy, and administrative talent necessary to the welfare of the army :—that officers with all the necessary qualifications can, however, be found, at all times, and in the required numbers, by another system—that of selection :—that public departments always take their tone from their heads, and that the efficiency with which business is carried on depends entirely on these heads :—that if discretion and energy be wanting at the head, the details of every branch must of necessity be badly conducted :—that it is so, and has long been so, in India :—that the Board of Madras was governed by such feelings that no person could have confidence :—that they showed no knowledge of their profession :—that in his time the members themselves were quite aware of all this :—that the entire system adopted by the Board was confessedly bad :—that when the Board did possess power and patronage it was used, never from public considerations, but from private views :—that men of worth and talent were neglected, while the idle, the careless, the most indifferent, and the most ignorant were protected and promoted :—that, in short, cringing, idleness, neglect, and ignorance had a better chance, under the Board, than talent and industry :—that, through this depraving system, he has known officers become "old Indians, with all their follies, before they had been two years in India :"—

F 2

that the chief boast of the members was the number of years they had lived in India:*—that so unworthy was the conduct of the Board, in the important matter of patronage, that this power was obliged to be withdrawn by a special and direct order from the Home Government:—that however respectable in private life some individual members, Boards constituted like those of India, never have, and never can command the confidence of the government, the respect of the services, or of the profession at large:—that in truth they degrade the profession, and injure the service:—that it is hoped the recent improvements in the pensions and in the mode of promotion, ordered by the Home Government, will prove beneficial, if the latter be vigorously and systematically acted on:—that whatever the Indian medical services may formerly have been, there were amongst them, and there are now in greater numbers, men of as high a character for talent and experience, as in any public service in the world:—that from such men as these the most efficient staff might at all times, and may now, be selected:—that it is only because men of an opposite character have hitherto been placed in the higher stations that the whole establishment has been contemned, underrated, and undervalued.

11. That, after a full and careful consideration of the whole subject, Mr. Annesley would recommend that the medical services of India be made immediately to approximate as nearly as possible, to that of Her Majesty's army:—that there should be one responsible head as Director General, with three secretaries, who should conduct the duties as follows:—one for general correspondence, checking commissariat supplies and indents, which would give ample employment to one officer—one for medical stores, medical accounts and expenditures—and one for business purely professional, including the statistics of the army, medical topography, &c.;—that it is now unnecessary to notice how these various and necessary duties have hitherto been performed by one secretary at each of the Presidencies:—that the cost of the Board, as now constituted at Madras, is as follows:—

| | Rupees. |
|---|---|
| Three Members at 2,450 rupees per Mensem.. .. | 7,350 |
| One Secretary at 700 ,, ,, .. .. | 700 |
| | 8,050:— |

that Mr. Annesley would propose, instead of the above plan, the following for the Madras Presidency:—

| | Rupees. |
|---|---|
| A Director General, at per Mensem .. .. .. | 3,000 |
| Three Secretaries, at per Mensem, 700 rupees each | 2,100 |
| | 5,100:— |

that by such an arrangement the efficiency of the department would be

* A member of the Bengal Board, lately returned,—a genuine sample of the seniority system—boasted, to his fellow-passengers on the homeward voyage, that he had entered at the bung-hole and come out at the hawse-hole of the service, in the course of his forty-three years residence;—an elegant improvement this upon the Ab ovo usque ad mala of Horace!

enormously enhanced, while a saving would accrue of rs. 2950 per mensem of the public money:—that, in addition to a complete regimental medical staff of one surgeon and one assistant to every native battalion of one thousand men, there should be for every division of an army in the field, one deputy inspector, two staff surgeons, three staff assistant surgeons, three apothecaries, four assistant apothecaries, and five dressers:—that ten doolies should be attached to the medical staff here cited, for the use of head-quarters, sick officers, rear guards, &c.:—that eight hospital tents should also be attached to the same staff, quite independently of the usual supply of sick tents supplied from the line:—that a medical store-keeper with complete establishment be also attached to the above staff, which officer should never be employed on extra duties, but confined exclusively to his proper duties:—that a staff so arranged, and being well selected, would be perfect for a division of ten thousand men:—that a want of selection in officers, and in proper field arrangement has, to his personal knowledge, proved a serious impediment in Indian campaigns, and a source of grievous loss of life, just as in other parts of the world:—that, under an intelligent and energetic head, such establishment as is here described will be found to meet every emergency:—that all the sick carriage, and its order of march, should be under the orders of the chief medical staff, who will so arrange that not a sick man, woman, or child, is ever found to encumber the rear-guard:—that it will be easy to apportion the number and the grades of medical staff to the existing divisions of the Indian army in cantonments, holding in view their constant liability to immediate field service.

Such is the evidence afforded to us in the documentary and verbal forms by Mr. Annesley—an officer who had the almost singular distinction of passing with public honour through every grade of the Indian medical service,—even unto the "Board"—who served in the campaigns of 1801-2-3 and 4, in the South of India—at the capture and occupation of Java, as surgeon to H. M. 78th regiment, and subsequently as Staff Surgeon on that island—who served as Staff Surgeon to the Army of Reserve in 1815—who served as chief of the Medical Staff to the 1st and 3rd divisions of the Army of the South of India, in 1817-18 and 19, up to the battle of Mahidpore, and the capture of Talnair—whose eminent services were acknowledged in the most public manner by the Governments of India at home and abroad, as well as by the Lords Commissioners of the Admiralty—who terminated a career of uncommon activity and usefulness by being placed in the Medical Board of Madras—a position the least satisfactory to himself, or congenial to his ardent and enterprising disposition, of any he ever held;—indeed, the only office in which he found it difficult to serve creditably and usefully—a gentleman of the highest reputation for honor and integrity—an officer who stands in the foremost ranks of tropical military physicians—an approved author, a man of energetic mind, of enlarged and liberal views.

Perhaps we may hear the testimony of this officer objected to by persons who have passed their lives in extra-professional or engrossing civil occupations, or by poetical and suicidal secretaries to medical boards, or by respectable old gentlemen whose acquaintance with armies has not gone beyond the smooth glacis of Fort William and Fort St. George; but these gentlemen, and their notions we shall notice only, by laughing at them. We leave them to die, along with the "Boards," of their own absurdity; while we again laugh at their groans over the improved and improving knowledge and spirit of the times.

13. We find again that, while a medical board in Bengal was wrangling about red coats bound in black velvet—about white pantaloons, cocked-hats and black feathers—a senior member of a quondam Madras board was occupied in much the same dignified manner. "Would it be credited," says Mr. Annesley, "and yet it is very true that, the first member of the medical board actually made a remonstrance that the profession felt degraded, because the superintending surgeons were not allowed to wear a gold button-hole." Mr. Annesley declares that, in his time, rank was estimated by the members of the board, not by the professional dignity it conferred, or the position in the army it commanded, but by the high and important considerations of the position in the official circles of Madras it would secure to their wives, and the supreme honor of seeing these ladies handed to dinner "by a member of council, or senior merchant!"

It is needless for us again to refer to the character and merits of Mr. Annesley; and we very much regret that an undue delicacy towards a system he knew to be bad, and towards persons whom he considered to be useless, should, during so many years past, have deterred that distinguished officer from dealing with, and exposing this vile system, and these official abuses, in the searching, unsparing, public manner, that could alone put them down, and put an end to them. He has done it now, however, and done it well.

14. The "Boards," whether in the East or the West, in the North or the South, are exact types of each other, and of that miserable system of organised neglect which, for sixty years, has marred the moral development and professional advancement of the Indian medical establishments, so far as "Boards" could mar a most able body of officers—a system that has never gone beyond the cut of the coat, or deeper than the skin of the wearer—a system that has never imagined a commencement at the heart, and a natural progress outwards—a system so miserable as to occasion the wonder no less than the contempt of every man capable of reflection—a system, however, which is now rapidly drawing to an end—*deploratus de medicis ac destitutus.*

15. Of the three grand requisites to efficient medical administration—a rapid promotion—a selection of staff officers—an experienced, able, vigorous and personal superintendence and control—we have absolutely none in India. This we have proved in every page—almost in every sentence, we have written on the subject.

Lastly. We would do the Governments of India, both at home and abroad, the justice to declare emphatically that, for the shameful neglect of the medical departments of the Indian army, they are not altogether responsible. A Government more considerate, just, and liberal, exists not, or one more disposed to place its servants on a footing of public and private respectability:—but, in the instance before us, the condition of the medical service, its neglected state, its wants, its disabilities, in all their relations, were never, until now, so far as we know, brought fully and fairly to the notice of authority. The services never united—the "Boards" never moved—nothing was ever said or complained of in the right quarter, in the right manner, or with a full and proper knowledge of the subject;—no wonder that nothing was done.* But we have now done with this subject. We entered upon it with reluctance. We took the open public mode of exposing abuses, only when other and milder modes have failed. We *have* exposed these abuses, in a way not to be gainsaid. We had no personal views or interests to serve. We have done substantive good, and we hope to do more. We have no ill-will towards any persons. We wish well to the Service.

* When Mr. Martin presented himself at the India House in support of the extension to his brother officers of the pension increasing according to length of service, and of the abolition of seniority as a ground for staff promotion—a ground as unwise and injurious in itself as opposed to the standing orders of the Bengal army—he was received with the utmost attention and consideration by every member of the Direction. His demands were just, and they were readily conceded.

It was another affair to obtain even a hearing from the Indian Minister of the Crown.

Here he had to contend with difficulties and delays of a nature impossible here to relate, for a whole year; and when these had all been overcome, and the Minister satisfied, and ready to act, a change of parties in the State took place, whence another year, more delays and difficulties, and more explanations were required. The new Minister, however, like the former, satisfied himself of the entire justice of the demands made upon him; but it is not too much to say that, but for the laborious and persevering exertions of Mr. Martin, no boon whatever would have been obtained for many a long year to come.

